# Widespread EEG Changes Precede Focal Seizures

**DOI:** 10.1371/journal.pone.0080972

**Published:** 2013-11-19

**Authors:** Piero Perucca, François Dubeau, Jean Gotman

**Affiliations:** Montreal Neurological Institute, McGill University, Montreal, Quebec, Canada; University Paris 6, France

## Abstract

The process by which the brain transitions into an epileptic seizure is unknown. In this study, we investigated whether the transition to seizure is associated with changes in brain dynamics detectable in the wideband EEG, and whether differences exist across underlying pathologies. Depth electrode ictal EEG recordings from 40 consecutive patients with pharmacoresistant lesional focal epilepsy were low-pass filtered at 500 Hz and sampled at 2,000 Hz. Predefined EEG sections were selected immediately before (immediate preictal), and 30 seconds before the earliest EEG sign suggestive of seizure activity (baseline). Spectral analysis, visual inspection and discrete wavelet transform were used to detect standard (delta, theta, alpha, beta and gamma) and high-frequency bands (ripples and fast ripples). At the group level, each EEG frequency band activity increased significantly from baseline to the immediate preictal section, mostly in a progressive manner and independently of any modification in the state of vigilance. Preictal increases in each frequency band activity were widespread, being observed in the seizure-onset zone and lesional tissue, as well as in remote regions. These changes occurred in all the investigated pathologies (mesial temporal atrophy/sclerosis, local/regional cortical atrophy, and malformations of cortical development), but were more pronounced in mesial temporal atrophy/sclerosis. Our findings indicate that a brain state change with distinctive features, in the form of unidirectional changes across the entire EEG bandwidth, occurs immediately prior to seizure onset. We postulate that these changes might reflect a facilitating state of the brain which enables a susceptible region to generate seizures.

## Introduction

Epileptic seizures are sudden interruptions of normal brain function, which result from an abnormal discharge of a neuronal ensemble [1]. Growing evidence [2], [3], [4], [5], however, suggests that the transition into a seizure may not be abrupt. Subtle clinical [6], [7], metabolic [8] and electrophysiological [3], [9], [10], [11], [12], [13] changes preceding seizure onset rather support the existence of a state in which the brain will evolve into a seizure unless a disruptive intervention takes place [14]. Assessing the existence and defining the characteristics of this pre-seizure (preictal) state is therefore inherently relevant to improving the understanding of mechanisms of seizure generation. Moreover, this is a prerequisite for rendering the preictal state a target for novel therapies [Fisher, 2012 #32][15].

Earlier studies of the preictal state have been aimed at predicting seizures, looking for changes over minutes or hours [9], [16], [17], [18]. We propose here to study the transition to seizures from a different angle: are there changes in the electroencephalogram (EEG) in the seconds before seizures, which could indicate that a change is occurring in the brain from which a seizure could emerge? The purpose is not to attempt seizure prediction but rather to see if there is a facilitating state, even if such a state is not necessarily specific.

We investigated spatio-temporal changes in standard EEG frequency bands and high-frequency oscillations (HFOs) occurring at the transition to seizure in a large cohort of patients with pharmacoresistant focal epilepsy undergoing intracranial EEG investigations. We also assessed whether these changes differ across three underlying pathologies, i.e. mesial temporal atrophy/sclerosis (MTS), local/regional cortical atrophy (CA) and malformations of cortical development (MCD).

## Materials and Methods

### Ethics statement

This study was approved by the Montreal Neurological Institute and Hospital Research Ethics Board (REB). The REB acts in conformity with standards set forth by the Tri-Council Policy Statement and Ethical Conduct for Research Involving Humans (Canada), and by the Rules and Regulations of the Department of Health and Human Services and the Food and Drug Administration (US) governing human subjects research and functioning in a manner consistent with internationally accepted principles of good clinical practice. All adult subjects signed an REB-approved written informed consent form. For subjects aged <18 years, written informed consent was obtained from the parents.

### Patients

Between November 2004 and May 2011, 101 patients underwent intracerebral depth electrode EEG investigations at a 2,000 Hz sampling rate. The indication for these investigations was to evaluate the feasibility of epilepsy surgery. From this sample, we enrolled all patients meeting the following criteria: a) MRI evidence of a focal lesion; b) recording of ≥1 seizures with EEG correlate; c) EEG sampling of lesional and non-lesional tissue; and d) EEG sampling of the seizure-onset zone (SOZ) and tissue outside the SOZ.

### Recording methods

Depth electrodes were implanted stereotactically using an image-guided system [19]. In most patients (82.5%), onsite-manufactured electrodes (9 contacts, 0.5–1 mm in length and 5 mm apart) [20] were used. In the remaining cases (17.5%), implantations were performed using DIXI Medical (Besançon, France) electrodes (5–18 contacts, 2 mm in length and 1.5 mm apart). Surface cortical electrodes [19] were also placed in a subgroup of patients (42.5%). The EEG was low-pass filtered at 500 Hz, sampled at 2,000 Hz, and recorded on Harmonie (Stellate, Canada). The recording was performed with a reference electrode placed over the parietal lobe contralateral to the suspected epileptogenic zone. EEG analyses were conducted using bipolar montages made from adjacent contacts. All contacts were analyzed, except for those outside the brain, non-functional or showing artifacts.

### Selection of seizures and EEG sections

For each patient, we assessed one representative seizure for each seizure type. Seizure types were defined by an experienced neurologist (F.D.) on the basis of electroclinical characteristics. Seizures with a clear EEG discharge but no apparent clinical manifestations (electrographic seizures) were considered a separate seizure type. For example, a patient with complex partial seizures and electrographic seizures arising from one temporal lobe was considered as having two seizure types. A seizure was analyzed only if separated by ≥1 hour from other seizures.

**Figure 1:**
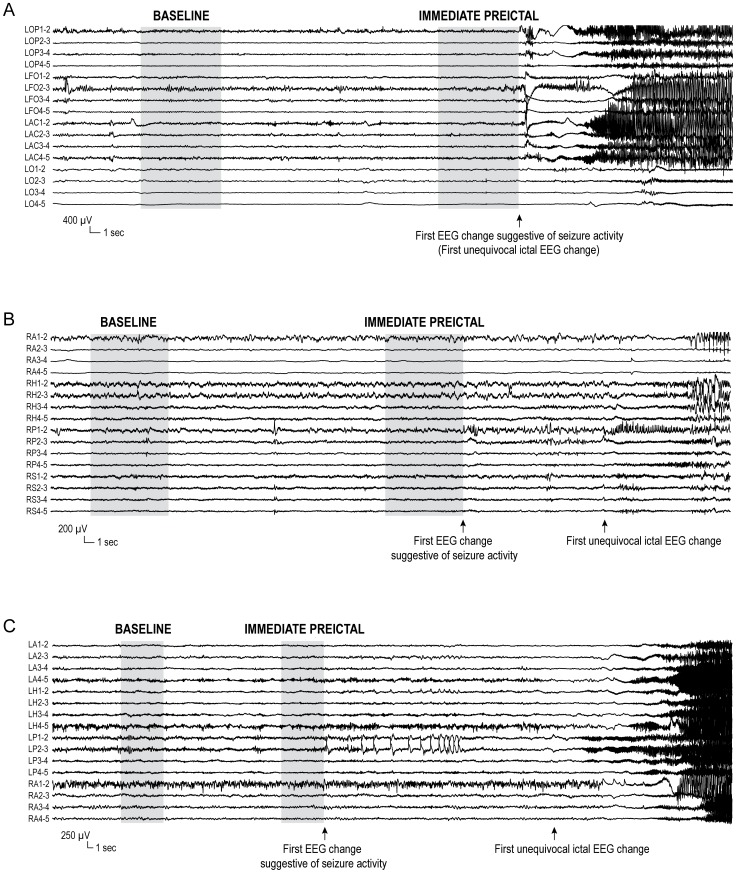
Definition of the “first EEG change suggestive of seizure activity”. This was the earliest potential ictal EEG sign, and could coincide with or precede the first unequivocal ictal EEG change. (A) Seizure recorded in a patient with a left centro-parietal porencephalic cyst, in which the sudden onset of polyspike/fast activity at LOP and LFO (arrow) was not preceded by any ictal-like EEG change. (B) Seizure recorded in a patient with right posterior quadrant periventricular nodular heterotopia, in which the first unequivocal ictal EEG change, a build-up of polyspikes at RP1-2 and 2-3 (arrow), was preceded 14 seconds earlier by the insidious appearance of polymorphic slowing with intermingled spikes at the same contacts (first EEG change suggestive of seizure activity, arrow). (C) Seizure recorded in a patient with left mesial temporal atrophy, in which the first unequivocal ictal EEG change, low-voltage fast activity at LP1-2 and 2-3 (arrow), was preceded 43 seconds earlier by the appearance of a run of repetitive sharp waves at the same contacts, which was not followed by a return to the usual EEG background (first EEG change suggestive of seizure activity, arrow). LOP = left frontal operculum; LFO = left orbito-frontal region; LAC =  left anterior cingulate gyrus; LO = left occipito-parietal region; RA = right amygdala; RH = right hippocampus; RP = heterotopic nodule in the right temporo-occipital quadrant; RS = heterotopic nodule in the right inferior parietal region; LA = left amygdala; LH = left hippocampus; LP = left posterior hippocampus.

**Figure 2:**
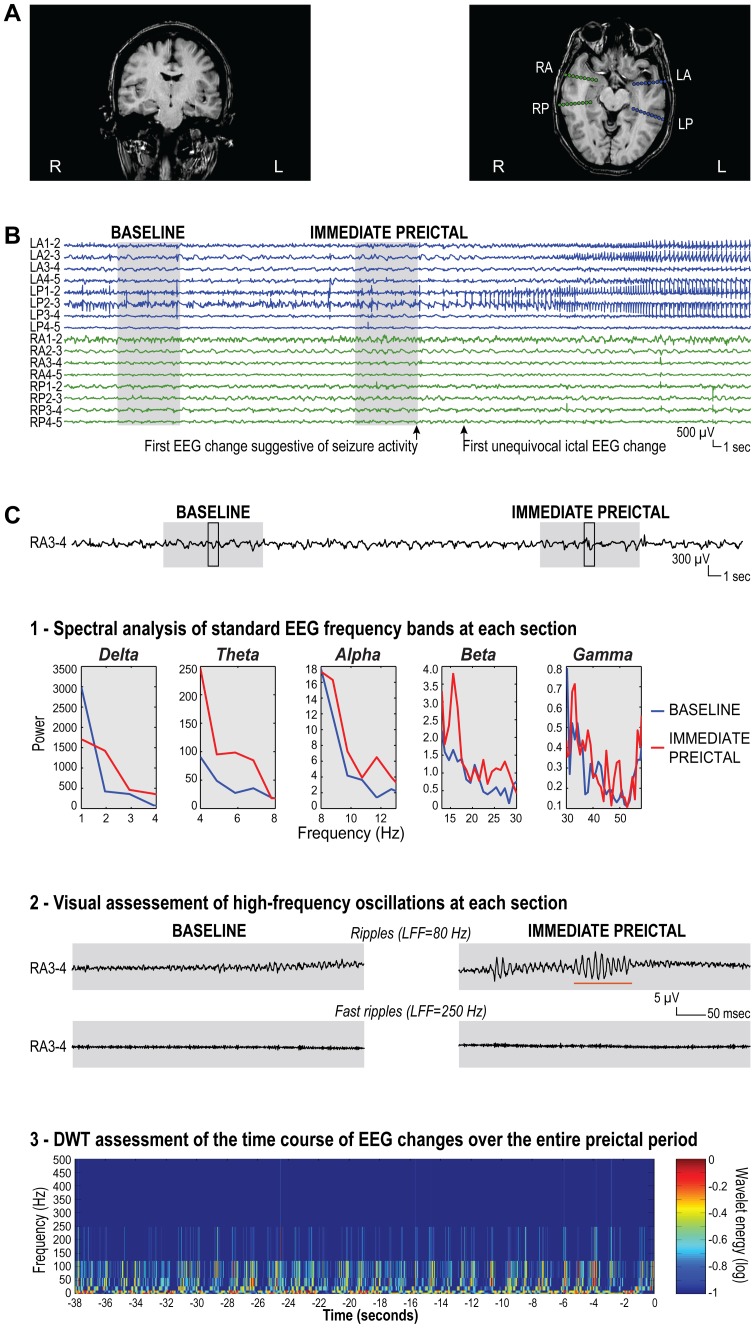
Assessment of preictal EEG changes in a patient with left mesial temporal atrophy. (A) MRI images showing marked volume loss in the left hippocampal formation (coronal T1-weighted image), and the depth electrode implantation sites (amygdala and hippocampus on each side) aimed at ruling out the possibility of bilateral independent temporal lobe seizure foci (axial T1-weighted image). (B) Seizure in which the first unequivocal ictal EEG change, a build-up of medium-to-high amplitude polypsikes at LP1-2, 2-3 (arrow), was preceded 6 seconds earlier by an insidious increase in spiking activity at the same contacts (first EEG change suggestive of seizure activity, arrow). (C) Assessment of different EEG frequency bands at contacts RA3-4, which were located in the hemisphere contralateral to the seizure-onset zone. This was performed *at each section* and *over the entire period extending from one section to the other*: 1) identification of standard EEG frequency bands (delta, theta, alpha, beta and gamma) at each section using spectral analysis. Note the increase in spectral power across all bands from the baseline (blue line) to the immediate preictal section (red line). These increases in power, which were small in magnitude, were virtually undetectable by visual inspection of the raw EEG signal; 2) visual analysis of high-frequency oscillations at each section, after extension of the time scale and high-pass filtering at 80 Hz (ripples) and 250 Hz (fast ripples). The EEGs shown here correspond to the segment of the original EEG signal included in the black-framed box within each section. Note that, while no ripple was found in the baseline, occasional ripples were observed in the immediate preictal section (as indicated by the orange underscore). No fast ripple was found in either section; 3) assessment of the time course of EEG changes over the entire period extending from the beginning of the baseline to the end of the immediate preictal section using discrete wavelet transform. Preictal changes were not clearly visible on the time-frequency map. R = right; L = left; RA = right amygdala; RP = right posterior hippocampus; LA = left amygdala; LP = left posterior hippocampus; LFF = low-frequency filter; DWT = discrete wavelet transform.

For each seizure, the “first EEG change suggestive of seizure activity” was defined as the earliest EEG sign considered to be potentially related to ictal activity. As shown in Figures 1–2, this change could coincide with or precede by several seconds the first unequivocal ictal EEG change, which was used to define the SOZ (see below). Although these assessments were carried out using bipolar montages, initial ictal EEG changes could also be detected with a referential montage (Figure S1). Two characteristic 8-second sections were then selected from the unfiltered EEG (Figures 1–2): *immediate preictal*, ending immediately before the first EEG change suggestive of seizure activity; *baseline*, ending 30 seconds prior to the first EEG change suggestive of seizure activity. If an isolated large spike occurred within the baseline, this section was shifted backward or forward by a few seconds to exclude the spike.

### Assessment of frequency bands

**At each section.** Spectral analysis using fast Fourier transformation was applied to each section to identify standard frequency bands, namely: delta, 1–4 Hz; theta, 4–8 Hz; alpha, 8–13 Hz; beta, 13–30 Hz; and gamma, 30–58 Hz (Figure 2). Frequencies >58 Hz were excluded because of possible contamination by the main power source. The spectral power in each band was computed for each bipolar channel, using consecutive 1.024-second epochs, overlapping by 25%, averaged over each section (Welch’s method with a cosine window).

High-frequency oscillations (HFOs) were assessed visually (Figure 2) with a procedure used in multiple earlier studies [21]. In brief, contacts of the bipolar montage were displayed with the maximum time resolution of the computer monitor (∼0.6 seconds, 1,200 samples). The EEG was digitally high-pass filtered at 80 Hz and 250 Hz using a finite impulse response (FIR) filter to minimize ringing. The computer display was split vertically to allow inspection of the expanded EEG at each of the two filter settings simultaneously, with the 80 Hz high-pass filter on the left side and the 250 Hz high-pass filter on the right. An event was considered as a ripple if visible on the left side of the display (80 Hz) and not on the right (250 Hz). A fast ripple was marked if an event was visible on the right (250 Hz). Only discrete events containing ≥4 consecutive oscillations were regarded as HFOs, and two events were considered distinct if separated by ≥2 non-HFO oscillations. To account for the frequency of occurrence and the duration of individual events, the percentage of time occupied by HFOs in each section was computed for each bipolar channel, as done previously [22].

HFOs have been assessed mainly using visual inspection or wavelet analysis [23]. A prior study [20] demonstrated that using the spectral content of high-frequency bands is generally less proficient than visual inspection in identifying HFOs. This is attributable to HFOs being events of low rate of occurrence, low amplitude, and often short duration (10–30 ms). Even when HFOs are >50 ms, spectral band analysis is unable to consistently detect these events [20]. Therefore, in our study, spectral analysis was limited to the assessment of standard frequency bands.

**Over the entire preictal period extending from one section to the other.** To characterize the time course of EEG changes from one section to the other, we also applied discrete wavelet transform to the entire period extending from the beginning of the baseline to the end of the immediate preictal section (Figure 2). Discrete wavelet transform offers the advantage of decomposing the EEG signal into frequency bands similar to those traditionally used in EEG analysis and bands used to define HFOs: 1–2 Hz and 2–4 Hz (delta); 4–8 Hz (theta); 8–16 Hz (∼alpha); 16–31 Hz (∼beta); 31–62 Hz (∼gamma); 62–125 Hz and 125–250 Hz (∼ripples); 250–500 Hz (fast ripples). To facilitate interpretation, we combined 1–2 Hz and 2–4 Hz into one single band labeled as “delta”, and 62–125 Hz and 125–250 Hz into one labeled as “ripples”. The wavelet energy was computed for each bipolar channel, and averaged over 1-second epochs.

### Classification of contacts

Contacts were classified as lesional if located within the borders of the lesion as determined on the post-implantation MRI. If this was unavailable, lesional contacts were identified by combining the information from the reconstructed position of the electrodes from the Neuronavigation system [19], a post-implantation CT and a post-explantation MRI.

The SOZ was defined as the contacts showing the first unequivocal ictal EEG change during the intracranial investigation. This was conducted as part of the clinical investigation, independently of this study.

### Statistical analysis

Frequency band data (spectral band power, wavelet band energy and percentage of time occupied by HFOs) were log-transformed to approximate a normal distribution. To prevent results from being heavily influenced by patients in whom a higher number of seizures was assessed, frequency band data were averaged across seizures in patients with >1 seizure. Data were then pooled across patients.

The Student paired t-test was used to compare spectral band power and the percentage of time occupied by HFOs between baseline and immediate preictal sections in all contacts and in four contact subsets: lesional/SOZ; non-lesional/SOZ; lesional/non-SOZ; and non-lesional/non-SOZ. One-way analysis of variance (ANOVA) with the Scheffe test was used to detect significant between-group differences in frequency band changes across the four contact subsets. These analyses were performed in the entire patient sample and in three separate subgroups, defined according to the underlying pathology: MTS, CA and MCD. In patients with concomitant lesions, contacts inside lesions other than the one under investigation were excluded from the analyses.

Linear regression was used to determine the time course of changes in wavelet band energy (dependent variable) over the entire preictal period extending from one section to the other. This analysis was limited to all contacts in the entire patient sample and in the three patient subgroups.

The Simes procedure, a modified Bonferroni correction [24], was applied to each set of analysis to adjust for multiple comparisons, maintaining the level of significance at 0.05. All analyses were performed with SPSS 19.0 (IBM, Chicago, USA).

## Results

### Patient characteristics

A total of 40 patients were eligible for this study (Table 1). Almost half had MCD (focal cortical dysplasia, n = 10; periventricular nodular heterotopia, n = 4; polymicrogyria, n = 3; and tuberous sclerosis, n = 1), 15 (37.5%) had MTS, and 10 (25%) had CA. Three patients had concomitant MTS and CA.

**Table 1:** Demographic and clinical characteristics of the entire patient sample (n = 40).

Category	Subcategory	Value
Age, years, median (range)		36 (16–54)
Gender, n. (%)	Men	21 (52.5)
	Women	19 (47.5)
Duration of epilepsy, years, median (range)^a^		16 (2–51)
MRI-defined lesions, n. (%)^b^	Mesial temporal atrophy/sclerosis	15 (37.5)
	Local/regional cortical atrophy	10 (25)
	Focal cortical dysplasia	10 (25)
	Periventricular nodular heterotopia	4 (10)
	Polymicrogyria	3 (7.5)
	Tuberous sclerosis	1 (2.5)
Implanted depth electrodes, median (range)		6 (2–11)

a data missing for one patient; ^b^ 3 patients had concomitant mesial temporal atrophy/sclerosis and regional/local atrophy.

Seventy seizures were assessed (1 to 4 per patient). One seizure was assessed in 20 patients, two in 14 patients, three in 4 patients, and four in 3 patients. The mean (range) duration between the first EEG change suggestive of seizure activity (end of the immediate preictal section) and the first unequivocal ictal EEG change was 5 (0–43) seconds.

### Preictal changes in different frequency bands

**Figure 3:**
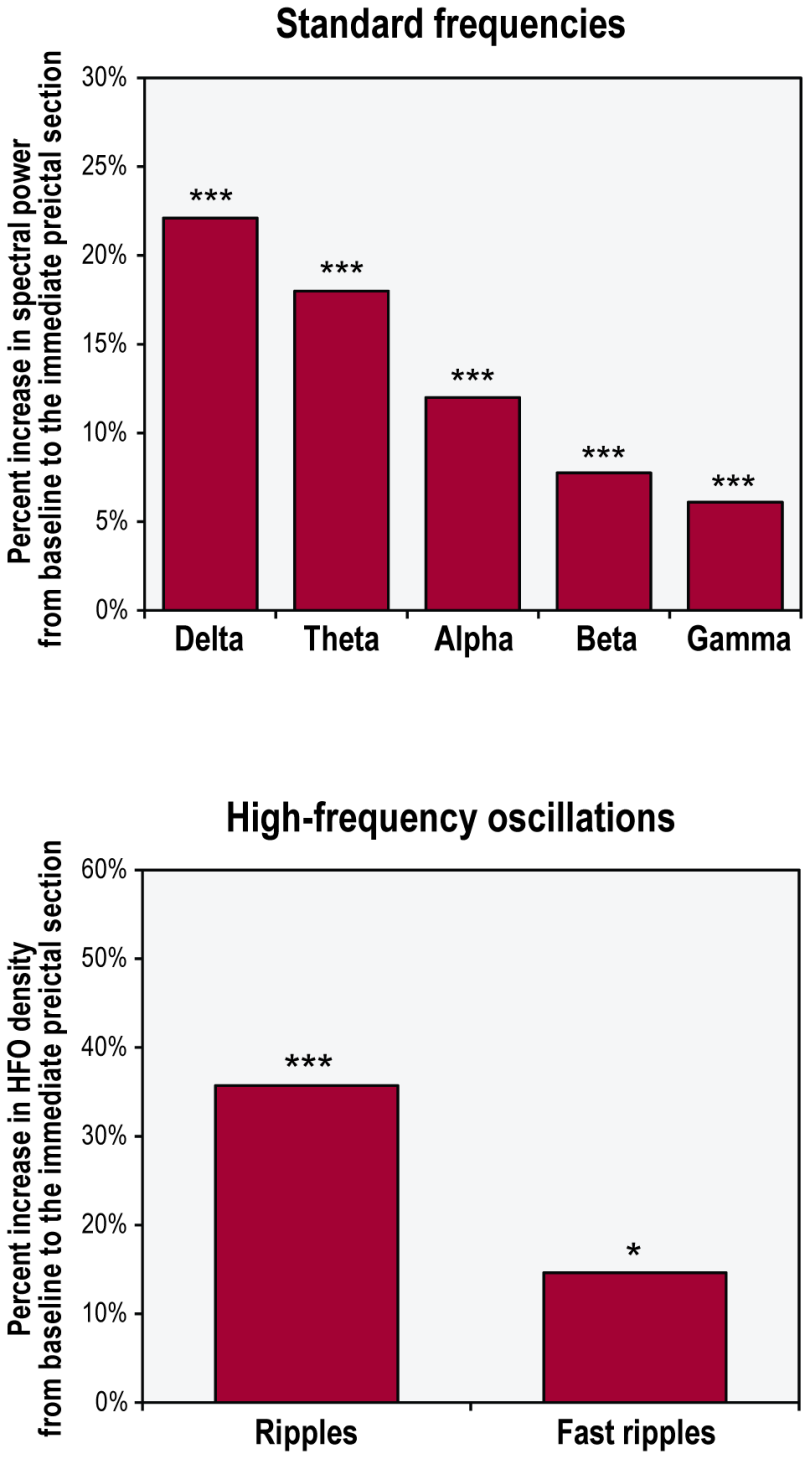
Preictal increases in the activity of different frequency bands in the entire patient sample. ***p<0.001; *p<0.05

**Figure 4:**
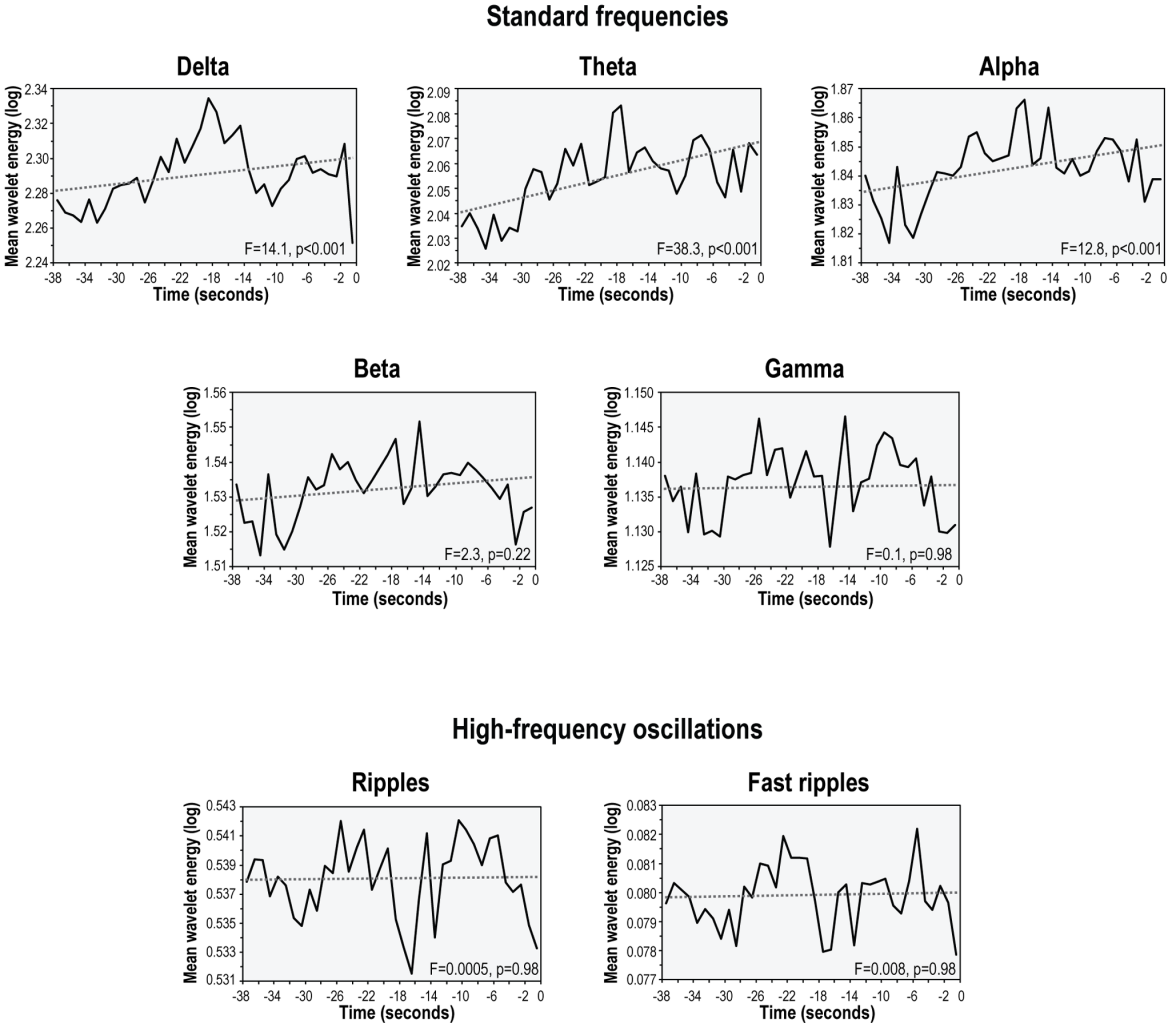
Time course of changes in the activity of different frequency bands over the entire preictal period extending from the beginning of the baseline to the end of the immediate preictal section in the entire patient sample. The dashed line in each graph indicates the linear regression line. Note the significant linear increase over time in wavelet energy for three of the seven frequency bands (delta, theta and alpha).

Each frequency band activity increased significantly from baseline to the immediate preictal section (Figure 3, Table S1). For standard frequency bands, the increase in spectral power ranged from 6.1% to 22.1%, with greater increases at lower frequencies. For high-frequency bands, the increase in the percentage of time occupied by HFOs was 35.7% for ripples and 14.6% for fast ripples. Over the entire period extending from the beginning of the baseline to end of the immediate preictal section, a linear increase in wavelet energy was found in three of the seven frequency bands (delta, theta and alpha; Figure 4).

Results were largely unchanged after excluding from the analyses seizures in which the baseline was shifted backward or forward by a few seconds to exclude isolated large spikes (n = 16, see Methods above), seizures in which clinical manifestations preceded the first EEG change suggestive of seizure activity (n = 4), or seizures in which a change in the state of vigilance from baseline to the immediate preictal section, clearly occurred or could not be excluded (n = 18), as determined by videotape review [25](data not shown). To rule out that the identified changes were attributable to a preictal increase in spiking, we re-analyzed the data after excluding electrode contacts with spikes (11.1% of all analyzed contacts). Since there is no universal definition of spikes, we defined them by visual examination, as would be done for clinical interpretation. As expected, the magnitude of the changes decreased slightly, but results remained significant, except for fast ripples (Figures S2–S3).

In a separate analysis, we investigated whether any of the identified preictal changes could be detected in individual seizures (Table S2). Of the 70 seizures included in the study, 58 (82.2%) were accompanied by a significant change in the activity of ≥1 frequency bands from baseline to the immediate preictal section. About two-thirds of these seizures were associated with only significant preictal increases in frequency band activity (36 seizures), 12 with both increases and decreases, and 10 with only decreases.

### Preictal changes in different frequency bands in relation to lesional tissue and the SOZ

**Figure 5:**
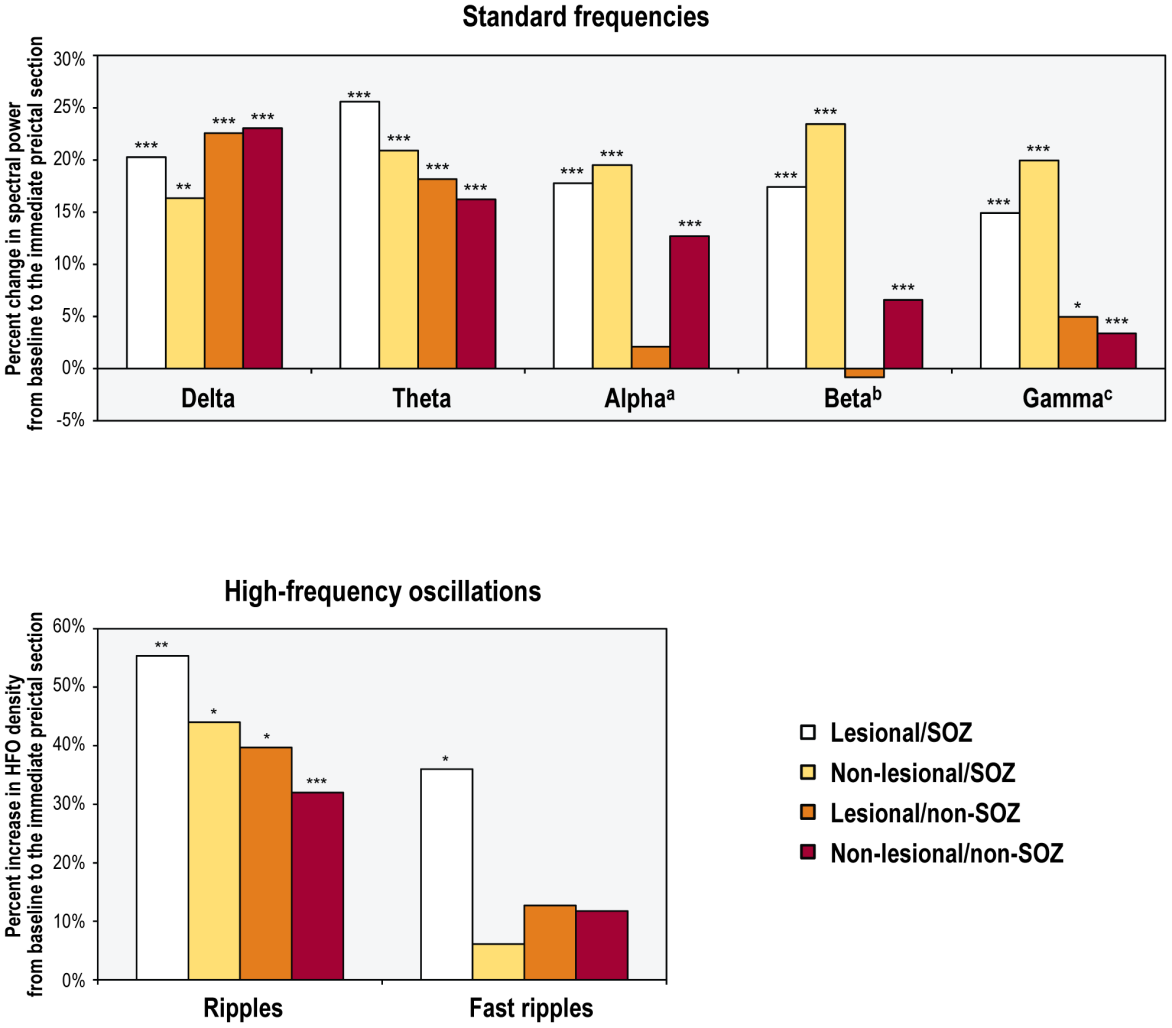
Preictal changes in the activity of different frequency bands across four separate contact subsets in the entire patient sample. ***p<0.001; **p<0.01; *p<0.05; (a) the preictal change in lesional/SOZ contacts was significantly different from that in lesional/non-SOZ contacts (Scheffe test; p<0.05); (b) the preictal change in lesional/SOZ contacts was significantly different from that in lesional/non-SOZ contacts, and from that in non-lesional/non-SOZ contacts (Scheffe test; p<0.001 and p<0.05, respectively). The preictal change in non-lesional/SOZ contacts was significantly different from that in lesional/non-SOZ contacts, and from that in non-lesional/non-SOZ contacts (Scheffe test; p<0.001 and p<0.01, respectively); (c) the preictal change in lesional/SOZ contacts was significantly different from that in lesional/non-SOZ contacts, and from that in non-lesional/non-SOZ contacts (Scheffe test; p<0.05 and p<0.01, respectively). The preictal change in non-lesional/SOZ contacts was significantly different from that in lesional/non-SOZ contacts, and from that in non-lesional/non-SOZ contacts (Scheffe test; p<0.01 and p<0.001, respectively).

The preictal increase in each frequency band activity was widespread, including non-lesional/non-SOZ contacts (Figure 5, Table S1). For most frequencies (delta, theta, alpha, and HFOs), there were no significant between-group differences in preictal changes across the four contact subsets, except for a greater preictal increase in alpha activity in lesional/SOZ contacts compared to lesional/non-SOZ contacts (p<0.05). For two frequency bands (beta and gamma), preictal changes inside the SOZ were greater than those outside, independent of whether the underlying tissue was lesional or not (all p<0.05).

### Effect of underlying pathology

**Figure 6:**
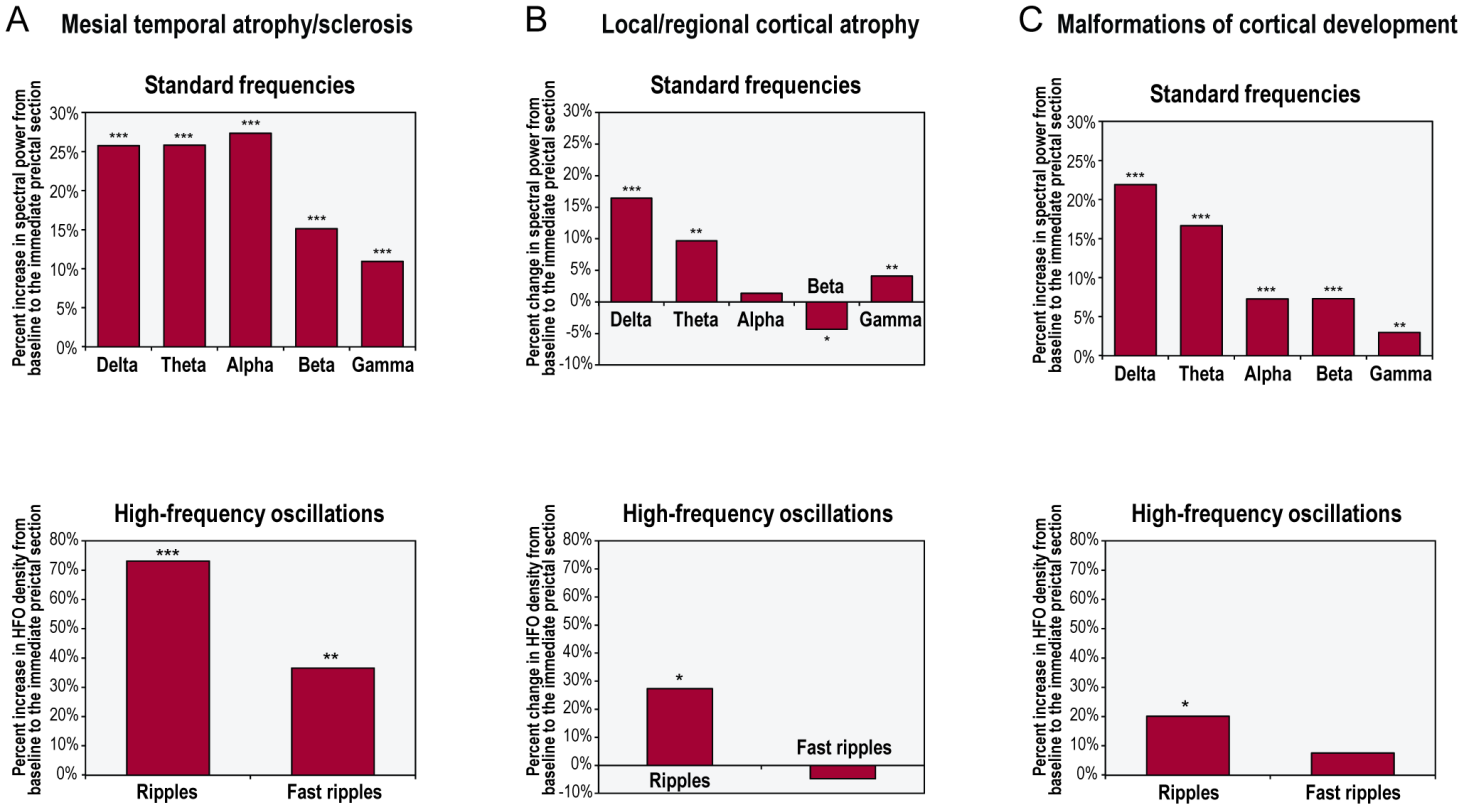
Preictal changes in the activity of different frequency bands in the three separate patient subgroups. The groups were defined according to the underlying pathology: (A) mesial temporal atrophy/sclerosis (n = 15); (B) local/regional cortical atrophy (n = 10); and (C) malformations of cortical development (n = 18). ***p<0.001; **p<0.01; *p<0.05.

**Mesial temporal atrophy/sclerosis.** Similarly to the entire sample, there was a significant preictal increase in each frequency band activity in the MTS subgroup (n = 15; Figure 6A, Table S3). For standard frequencies, increases in spectral power ranged from 10.9% (gamma) to 27.4% (alpha). For high frequencies, increases in the percentage of time occupied by HFOs were 73.1% for ripples and 36.6% for fast ripples. Over the entire period extending from the beginning of the baseline to end of the immediate preictal section, a linear increase in wavelet energy was found in three bands (delta, theta and alpha; Figure S4).

Preictal increases in each frequency band activity were again widespread, including non-lesional/non-SOZ contacts (Table 2; Table S3). For standard frequencies and ripples, there were no significant between-group differences in preictal changes across the four contact subsets, except for a greater preictal increase in theta activity in lesional/non-SOZ contacts and in gamma activity in non-lesional/SOZ contacts compared to non-lesional/non-SOZ contacts (p = 0.05 and p<0.01, respectively). For fast ripples, preictal changes in lesional/SOZ contacts were 44.9-fold greater than those in non-lesional/SOZ contacts, and 3.5-fold greater than those in non-lesional/non-SOZ contacts (both p<0.001).

**Table 2:** Preictal changes in the activity of different frequency bands across four separate contact subsets in the three patient subgroups.

		Percent change in frequency band activity from baseline to the immediate preictal section in four contact subsets			
Patient subgroup	Frequency band	Lesional/SOZ	Non-lesional/SOZ	Lesional/non-SOZ	Non-lesional/non-SOZ
Mesial temporal atrophy/sclerosis	Delta	27.9%**	25.0%**	52.8%***	23.7%***
	Theta^a^	39.1%***	30.6%**	59.0%**	21.5%***
	Alpha	36.8%***	38.8%***	37.9%**	24.2%***
	Beta	32.6%**	31.9%**	25.3%**	10.6%***
	Gamma^b^	18.5%*	33.7%**	17.2%	7.1%***
	Ripples	110.0%**	59.7%	159.1%	74.5%***
	Fast ripples^c^	157.1%**	–3.5%	41.9%	44.6%
Local/regional cortical atrophy	Delta	10.1%	15.2%	15.8%**	17.5%***
	Theta	–2.9%	8.3%	12.1%	10.0%**
	Alpha	–16.4%	13.5%	–6.7%	6.3%
	Beta^d^	–10.4%	27.3%	–13.1%**	–2.0%
	Gamma^e^	0.2%	23.7%*	4.3%	2.7%
	Ripples	41.2%	9.5%	19.8%	33.7%
	Fast ripples	–37.1%	–12.5%	5.4%	–2.4%
Malformations of cortical development	Delta	20.3%*	7.6%	21.6%**	23.9%***
	Theta	26.3%***	18.4%*	14.4%*	14.7%***
	Alpha	18.0%**	3.9%	2.1%	6.3%*
	Beta^f^	17.4%***	12.9%	4.8%	5.0%**
	Gamma^g^	16.4%**	4.2%	2.5%	–0.2%
	Ripples	39.3%	47.9%	32.0%	9.1%
	Fast ripples	9.6%	20.7%	8.5%	4.2%

*** p<0.001; **p<0.01; *p≤0.05.

a The preictal change in lesional/non-SOZ contacts was significantly different from that in non-lesional/non-SOZ contacts (Scheffe test; p = 0.05).

b The preictal change in non-lesional/SOZ contacts was significantly different from that in non-lesional/non-SOZ contacts (Scheffe test; p<0.01).

c The preictal change in lesional/SOZ contacts was significantly different from that in non-lesional/SOZ contacts, and from that in non-lesional/non-SOZ contacts (Scheffe test; both p<0.001).

d The preictal change in non-lesional/SOZ contacts was significantly different from that in lesional/SOZ contacts, from that in lesional/non-SOZ contacts, and from that in non-lesional/non-SOZ contacts (Scheffe test; p<0.05, p<0.01 and p<0.05, respectively).

e The preictal change in non-lesional/SOZ contacts was significantly different from that in non-lesional/non-SOZ contacts (Scheffe test; p<0.05).

f The preictal change in lesional/SOZ contacts was significantly different from that in non-lesional/non-SOZ contacts (Scheffe test; p<0.05).

g The preictal change in lesional/SOZ contacts was significantly different from that in lesional/non-SOZ contacts, and from that in non-lesional/non-SOZ contacts (Scheffe test; p<0.01 and p<0.001, respectively).

**Local/regional cortical atrophy.** In the CA subgroup (n = 10), a significant preictal increase was found in the activity of four of the seven frequency bands (delta, theta, gamma and ripples; Figure 6B, Table S3). For standard frequencies, increases in spectral power ranged from 4.1% (gamma) to 16.4% (delta). The increase in the percentage of time occupied by ripples was 27.4%. Of note, there was a slight, yet significant, preictal decrease in beta power (Figure 6B). Over the entire period extending from the beginning of the baseline to end of the immediate preictal section, a linear increase in wavelet energy was found only for theta (Figure S5).

Although more scattered than those found in the entire sample, preictal changes in this subgroup were again detected in non-lesional/non-SOZ contacts (Table 2; Table S3). For most frequency bands (delta, theta, alpha, and HFOs), there were no significant between-group differences in preictal changes across the four contact subsets. For the remaining frequency bands (beta and gamma), preictal changes in non-lesional/SOZ contacts were significantly greater than those in non-lesional/non-SOZ contacts (gamma) or those in any other contact subset (beta).

**Malformations of cortical development.** In the MCD subgroup (n = 18), there was a significant preictal increase in each frequency band activity, except for fast ripples (Figure 6C, Table S3). For standard frequencies, the increase in spectral power ranged from 3.0% to 21.9%, with greater increases at lower frequencies. The increase in the percentage of time occupied by ripples was 20.1%. Over the entire period extending from the beginning of the baseline to end of the immediate preictal section, a linear increase in wavelet energy was found only for theta (Figure S6).

Particularly for lower frequencies, preictal changes occurred in a widespread manner, involving non-lesional/non-SOZ contacts (Table 2; Table S3). For most frequency bands (delta, theta, alpha, and HFOs), no significant between-group differences in preictal changes were found across the four contact subsets. For the remaining frequency bands (beta and gamma), preictal changes in lesional/SOZ contacts were significantly greater than those in non-lesional/non-SOZ contacts (beta) or those in non-SOZ contacts, independent of whether the underlying tissue was lesional or not (gamma).

## Discussion

In this study, conducted in a large cohort of consecutively enrolled patients with pharmacoresistant lesional focal epilepsy undergoing intracranial EEG monitoring, we found that definite EEG changes occur in the seconds preceding the onset of focal seizures. These changes have two unexpected features: (i) they were observed across the entire EEG bandwidth, in the form of increases in the activity of standard frequency bands as well as HFOs; (ii) they exhibited a widespread topographical distribution, being detected in the SOZ and lesional tissue, as well as in remote regions. These results were reinforced by the analysis on separate pathologies. In fact, although the findings described above were more pronounced in MTS, all pathologies were associated with preictal EEG changes which were relatively independent of the type of tissue. Overall, these results indicate that the brain state appears to change slightly, in a diffuse manner prior to seizure onset.

To our knowledge, this is the first study to demonstrate that widespread changes in traditional EEG frequency bands and HFOs occur at the transition to seizure, irrespective of the underlying pathology. Preictal changes extending beyond the epileptogenic zone have also been observed by different means of brain function assessment [26], including chaos analysis of the EEG [9], [16], [27], measurement of cortical excitability with transcranial magnetic stimulation [11], and imaging-based assessment of cerebral haemodynamics [8], but all these were over a much longer time scale. The underlying mechanisms of our findings remain largely unknown. One hypothesis is that these changes reflect large-scale propagation of activity arising from the epileptogenic region, as it may occur during interictal discharges [9], [28], although not identifiable by traditional visual analysis. Alternatively, they could be the expression of a facilitating state of the brain, which enables a susceptible region to initiate seizure activity [9], [26]. This facilitating state may herald or trigger the emergence of glutamatergic preictal discharges in the SOZ, which based on recent *in situ* and *in vitro* data seem to be implicated in seizure generation in mesial temporal lobe epilepsy [3]. This brain state, on the other hand, could also be attributable to a systemic change, such as a pH imbalance. Systemic alkalosis can cause seizures [29], [30], whereas acidosis induced by inhaling 5%CO_2_ suppresses seizure activity in experimental models and patients with epilepsy [31]. In a recent study of patient electrocorticography recordings, brief oscillations of increasing frequency (from ∼30–40 Hz to >120 Hz) were found to emerge in the seconds preceding seizure onset, as a result of an alkaline fluctuation of the environmental pH [32]. These effects may be mediated by synaptic and non-synaptic transmission, including gap junctions, which are pH sensitive [32].

Another innovative aspect of our study was the control for confounders that can affect the identification of preictal EEG changes. First, to minimize the risk of including seizure activity in sections labeled “preictal”, we conservatively ended the selection immediately before the “first EEG change suggestive of seizure activity” (Figures 1–2). This early definition of the ictal onset preceded by an average of 5 seconds the “first unequivocal ictal EEG change”, which is commonly used to define the SOZ and the time of seizure onset [33], [34]. In a study in which the selection of the preictal section ended at the first unequivocal ictal EEG change, high-frequency activity increased in the SOZ approximately 8 seconds earlier. The changes being limited to the SOZ and the selection of the unequivocal onset raise the possibility that the “preictal” changes could be partly the expression of already ongoing seizure activity [35]. We also re-assessed preictal EEG changes after excluding seizures in which clinical manifestations preceded the electrographic onset, for which an earlier but unrecorded onset can be hypothesized, a sensitivity analysis which did not alter the results. Second, we investigated whether the identified preictal EEG changes could have reflected EEG changes accompanying transitions between states of vigilance, which are themselves associated with an increased risk of seizure occurrence [25], [36], [37]; this potential confounder is often neglected in EEG investigations of preictal phenomena [25]. Transitions between states of vigilance are typically accompanied by diverging changes in the EEG. The sleep-to-wakefulness transition, for instance, is characterized by attenuation of delta and theta activity, and enhancement of fast frequencies; HFOs, on the other hand, appear to decrease with wakefulness [38]. In our study, however, there was a significant increase across all frequency bands immediately prior to seizure onset, which argues against the possibility of EEG changes being secondary to changes in states of vigilance. Moreover, excluding from the analyses seizures in which a change in the state of vigilance clearly occurred before seizure onset, or could not be excluded, did not alter the results. Third, we assessed whether our findings could be explained by a preictal increase in spiking. Contrary to a long-standing assumption, increases in spiking before seizures have not been clearly documented [39], [40]. Even if spikes were to increase prior to seizure onset, they would be associated with changes in a broad range of frequencies limited to epileptogenic areas. In our study, increases in the activity across the entire EEG spectrum were found in all regions, including those that were remote from the SOZ and lesional tissue. Moreover, the exclusion of spiking contacts from the analysis of preictal changes modified the results only slightly.

It is unlikely that our findings are attributable to another factor linked with preictal changes, i.e. direct current (DC) shifts. DC shifts are very slow potentials (<1 Hz) which can precede by up to 10 seconds the first unequivocal ictal EEG change [41]. They tend to occur in sub-regions of the SOZ [41], [42]. In contrast, the changes identified in our study extended beyond slow frequencies and affected all regions investigated.

Preictal EEG changes were generally small in magnitude. For standard frequency bands, the increase in spectral power from baseline to the immediate preictal section ranged between 5% and 25%. This level of energy change can easily elude visual inspection (Figure 2), which may explain why similar observations have not been reported before. Nonetheless, these changes are robust as they were also detected in the smaller lesion-dependent patient subgroups. Future studies should investigate whether these phenomena can be captured over a larger time scale (in the minutes or hours prior to seizure occurrence) or even on scalp EEG recordings.

In conclusion, widespread changes in traditional and novel EEG hallmarks precede the onset of focal seizures, independently of modifications in state of vigilance and underlying pathology. Elucidating mechanisms underpinning these changes could lead to an improved understanding of the pathophysiology of seizures and epilepsies.

## Supporting Information

Figure S1 **Bipolar montage vs referential montage in the assessment of initial ictal EEG changes.** A seizure from a patient with right posterior quadrant periventricular nodular heterotopia (the same shown in Figure 1B) is displayed using a bipolar montage made from adjacent contacts (top) and a referential montage with the reference (LPar) placed over the left parietal lobe, which was contralateral to the suspected epileptogenic zone (bottom). Note that the “first EEG change suggestive of seizure activity” (polymorphic slowing with intermingled spikes at RP1, red arrows) and the “first unequivocal ictal EEG change” (build-up of polyspikes at RP1, black arrows) can be detected with both types of montages. RA = right amygdala; RH = right hippocampus; RP = heterotopic nodule in the right temporo-occipital quadrant; RS = heterotopic nodule in the right inferior parietal region; LPar = left parietal lobe.
(TIF)

Figure S2 **Preictal changes in the activity of different frequency bands in the entire patient sample, after exclusion of contacts with spikes.** Note the similarity of these findings with the original analysis (Figure 3). ***p<0.001; **p<0.01; *p<0.05.
(TIF)

Figure S3 **Time course of changes in the activity of different frequency bands over the entire preictal period extending from the beginning of the baseline to the end of the immediate preictal section in the entire patient sample, after exclusion of contacts with spikes.** The dashed line in each graph indicates the linear regression line. Note the significant linear increase over time in wavelet energy for three of the seven frequency bands (delta, theta and alpha), as in the original analysis (Figure 4).
(TIF)

Figure S4 **Time course of changes in the activity of different frequency bands over the entire preictal period extending from the beginning of the baseline to the end of the immediate preictal section in the subgroup with MTS.** The dashed line in each graph indicates the linear regression line. Note the significant linear increase over time in wavelet energy for three frequency bands (delta, theta and alpha).
(TIF)

Figure S5 **Time course of changes in the activity of different frequency bands over the entire preictal period extending from the beginning of the baseline to the end of the immediate preictal section in the subgroup with CA.** The dashed line in each graph indicates the linear regression line. Note the significant linear increase over time in wavelet energy for one frequency band (theta).
(TIF)

Figure S6 **Time course of changes in the activity of different frequency bands over the entire preictal period extending from the beginning of the baseline to the end of the immediate preictal section in the subgroup with MCD.** The dashed line in each graph indicates the linear regression line. Note the significant linear increase over time in wavelet energy for one frequency band (theta).
(TIF)

Table S1 **Comparison of the activity of different frequency bands between the baseline section and the immediate preictal section in the entire patient sample, shown for the entire set of contacts and for four separate contact subsets (lesional/SOZ, non-lesional/SOZ, lesional/non-SOZ, non-lesional/non-SOZ).** For standard frequency bands, values in cells are mean spectral power with 95% confidence interval (95% CI). For HFOs, values in cells are mean percentage of time occupied by HFOs in each section (95% CI).
(DOC)

Table S2 **Preictal changes in the activity of different frequency bands in each of the seizures included in the study (n = 70).** Seizures have been stratified according to the type of significant preictal changes in frequency band activity: increases only (36 seizures), both increases and decreases (12 seizures), decreases only (10 seizures), and no significant change (12 seizures).
(DOC)

Table S3 **Comparison of the activity of different frequency bands between the baseline section and the immediate preictal section in the three patient subgroups, shown for the entire set of contacts and for four separate contact subsets (lesional/SOZ, non-lesional/SOZ, lesional/non-SOZ, non-lesional/non-SOZ).** For standard frequency bands, values in cells are mean spectral power with 95% confidence interval (95% CI). For HFOs, values in cells are mean percentage of time occupied by HFOs in each section (95% CI).
(DOC)
