# Supplementary figures and images for: Widespread EEG Changes Precede Focal Seizures
Source: PLoS One. 2013 Nov 19;8(11):e80972. doi: 10.1371/journal.pone.0080972 (PMC3834227; doi:10.1371/journal.pone.0080972)

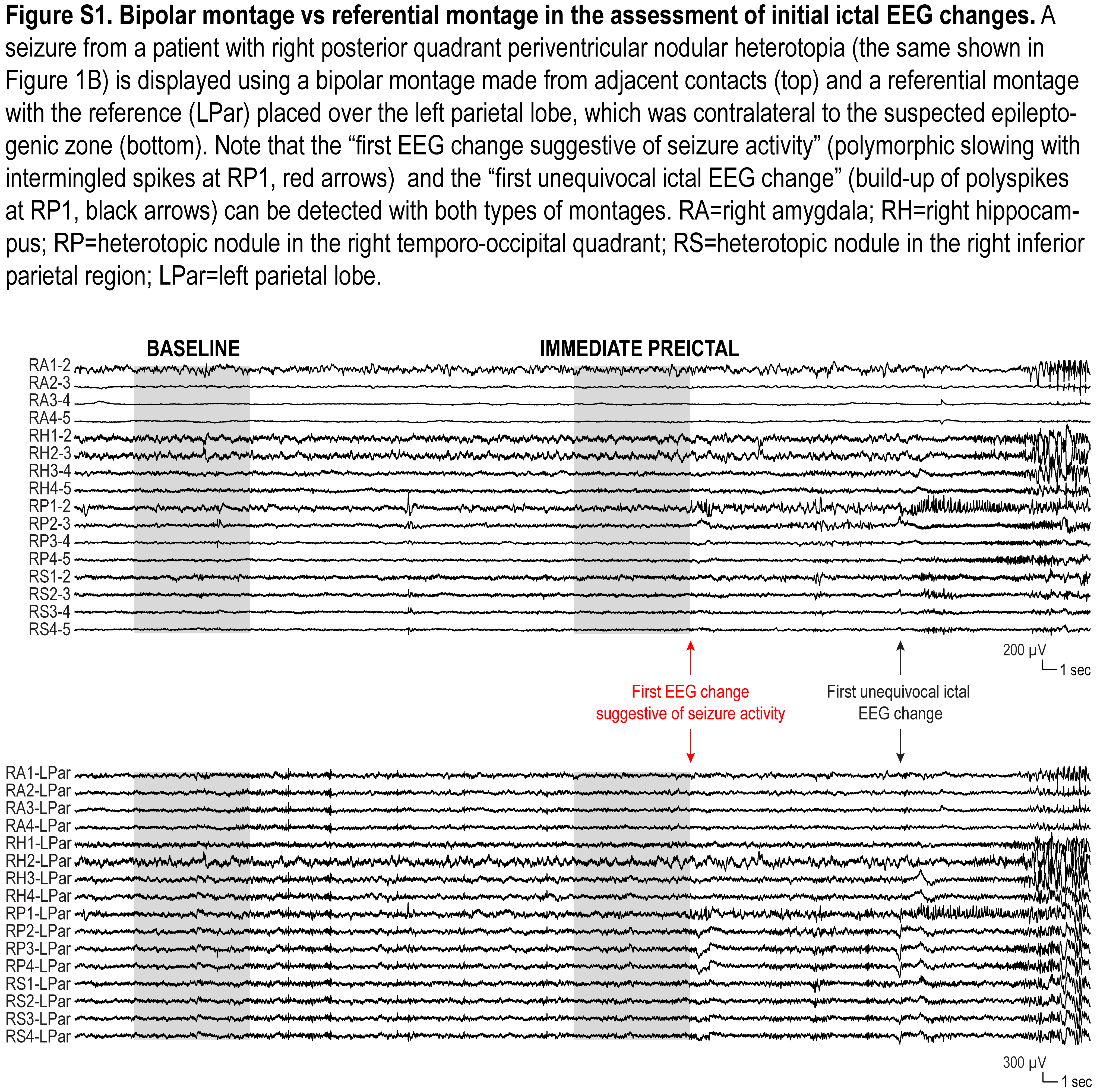

Supplement: Figure S1 — Bipolar montage vs referential montage in the assessment of initial ictal EEG changes. A seizure from a patient with right posterior quadrant periventricular nodular heterotopia (the same shown in Figure 1B) is displayed using a bipolar montage made from adjacent contacts (top) and a referential montage with the reference (LPar) placed over the left parietal lobe, which was contralateral to the suspected epileptogenic zone (bottom). Note that the “first EEG change suggestive of seizure activity” (polymorphic slowing with intermingled spikes at RP1, red arrows) and the “first unequivocal ictal EEG change” (build-up of polyspikes at RP1, black arrows) can be detected with both types of montages. RA = right amygdala; RH = right hippocampus; RP = heterotopic nodule in the right temporo-occipital quadrant; RS = heterotopic nodule in the right inferior parietal region; LPar = left parietal lobe. (TIF) [file pone.0080972.s001.tif]

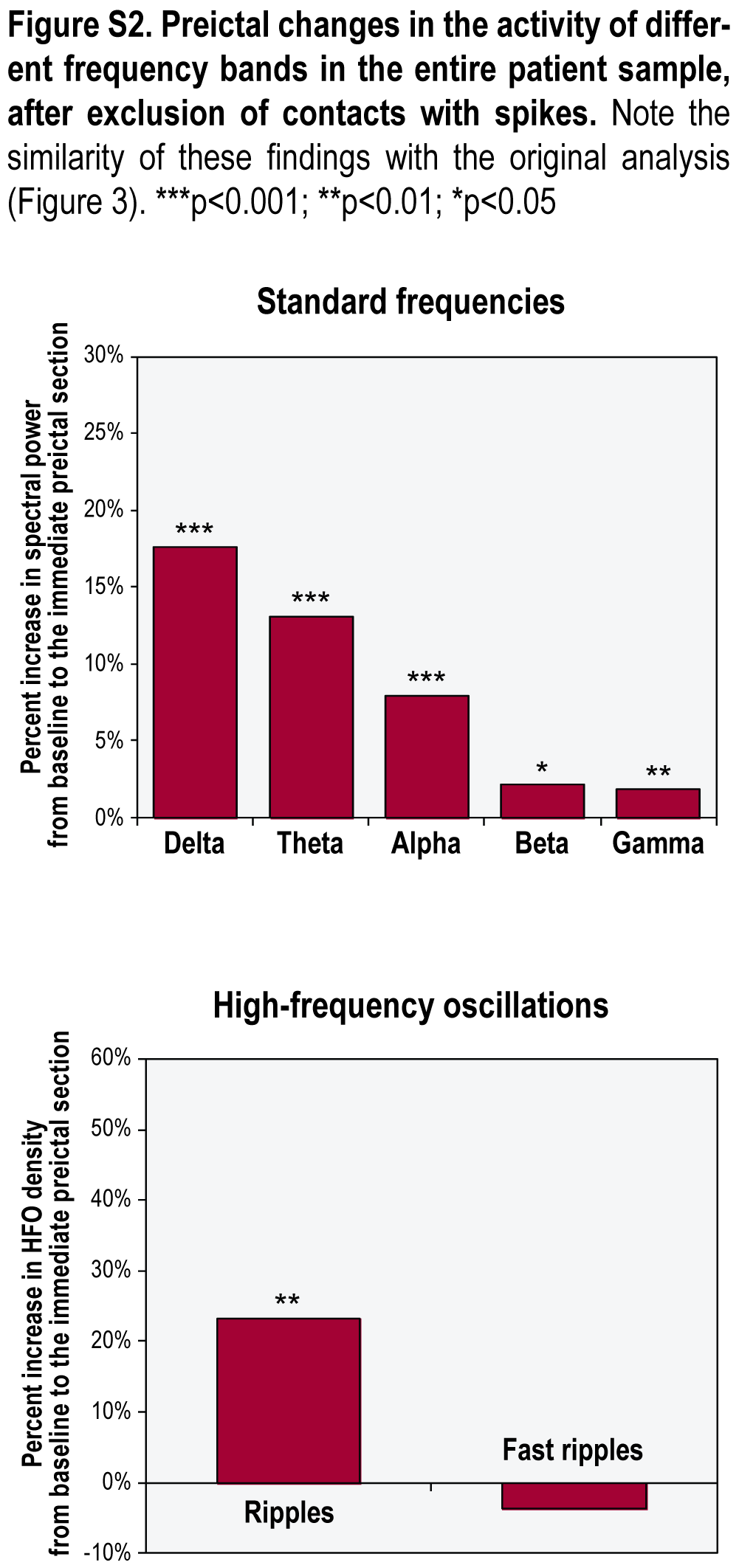

Supplement: Figure S2 — Preictal changes in the activity of different frequency bands in the entire patient sample, after exclusion of contacts with spikes. Note the similarity of these findings with the original analysis (Figure 3). ***p<0.001; **p<0.01; *p<0.05. (TIF) [file pone.0080972.s002.tif]

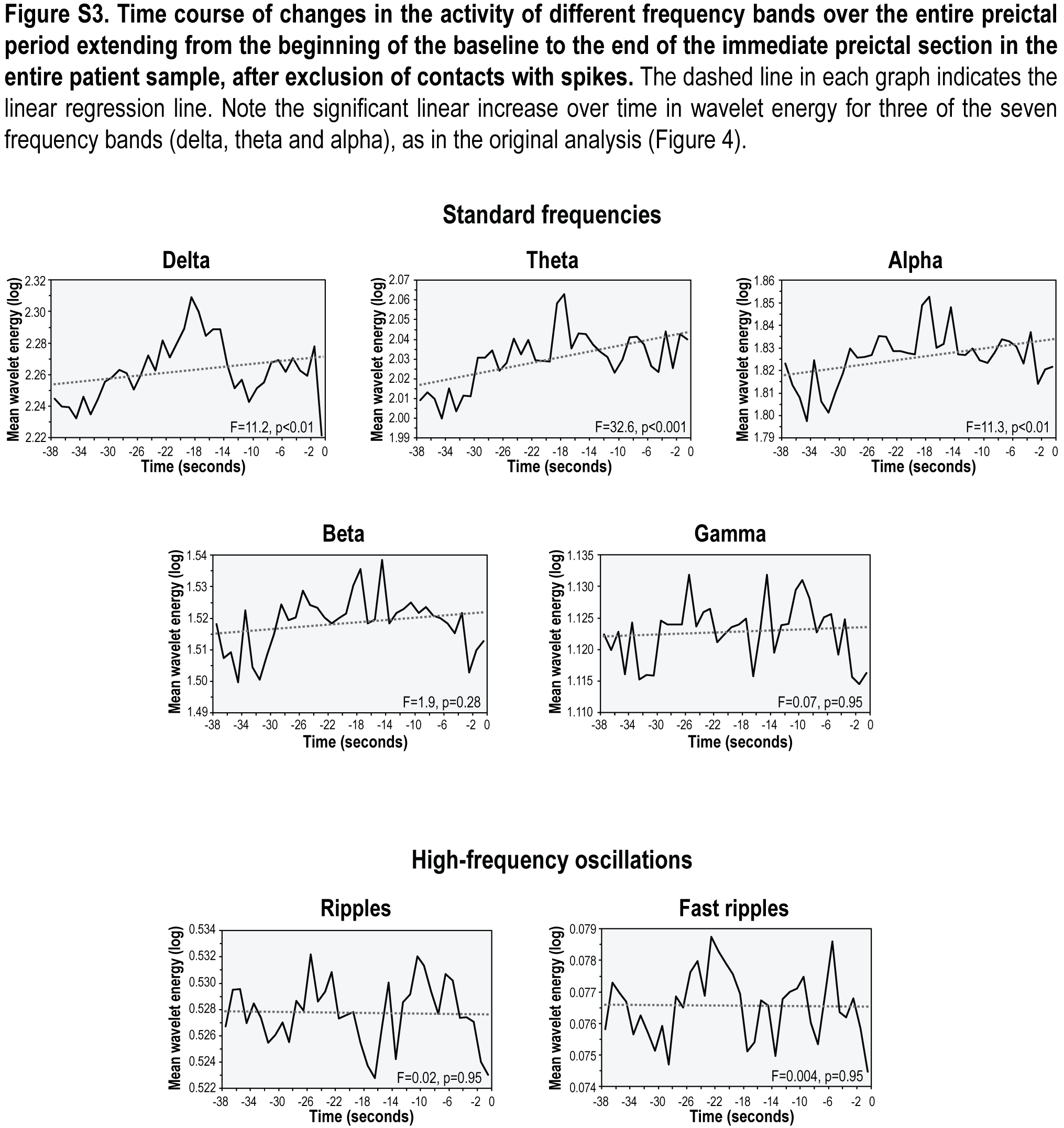

Supplement: Figure S3 — Time course of changes in the activity of different frequency bands over the entire preictal period extending from the beginning of the baseline to the end of the immediate preictal section in the entire patient sample, after exclusion of contacts with spikes. The dashed line in each graph indicates the linear regression line. Note the significant linear increase over time in wavelet energy for three of the seven frequency bands (delta, theta and alpha), as in the original analysis (Figure 4). (TIF) [file pone.0080972.s003.tif]

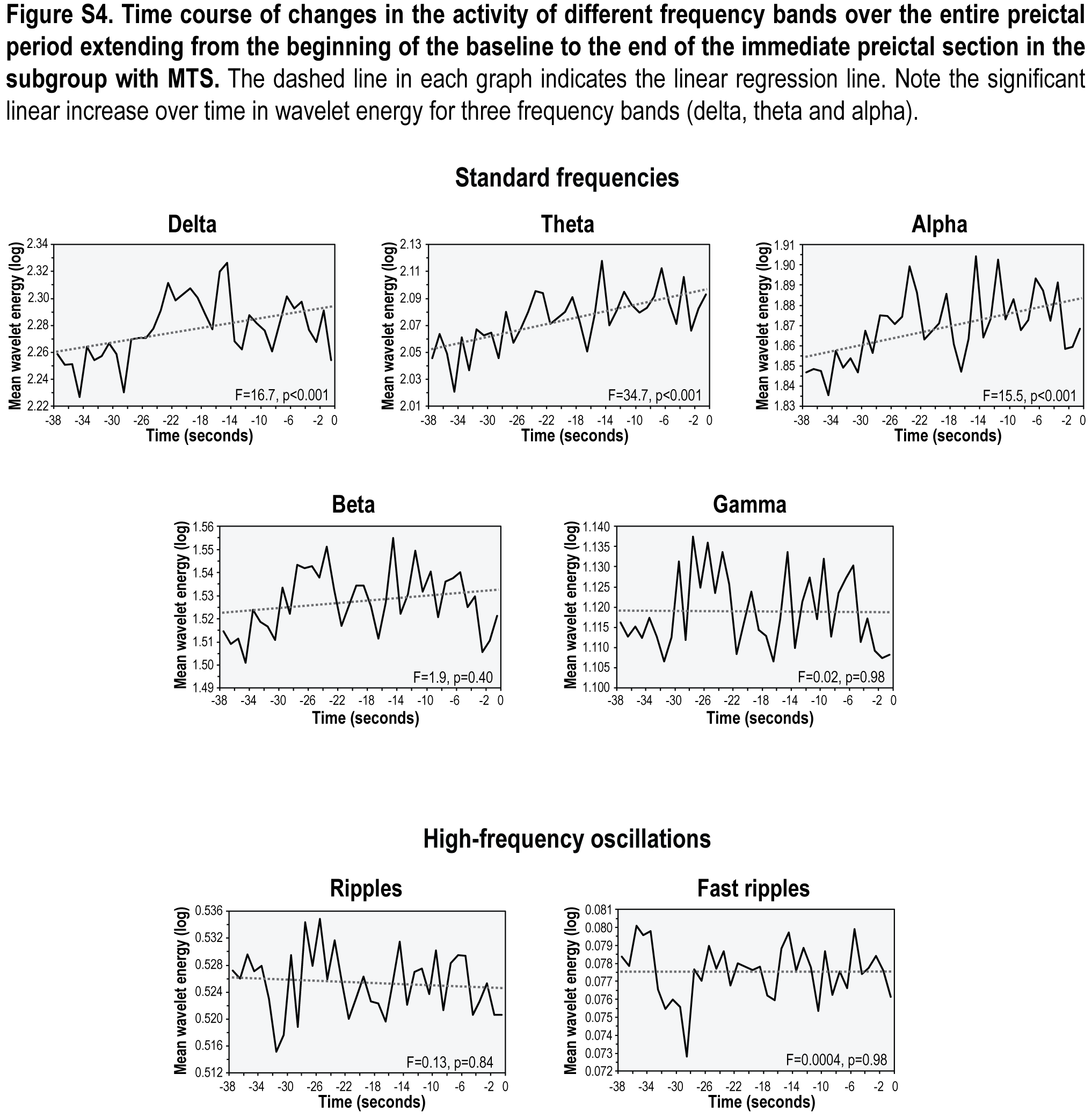

Supplement: Figure S4 — Time course of changes in the activity of different frequency bands over the entire preictal period extending from the beginning of the baseline to the end of the immediate preictal section in the subgroup with MTS. The dashed line in each graph indicates the linear regression line. Note the significant linear increase over time in wavelet energy for three frequency bands (delta, theta and alpha). (TIF) [file pone.0080972.s004.tif]

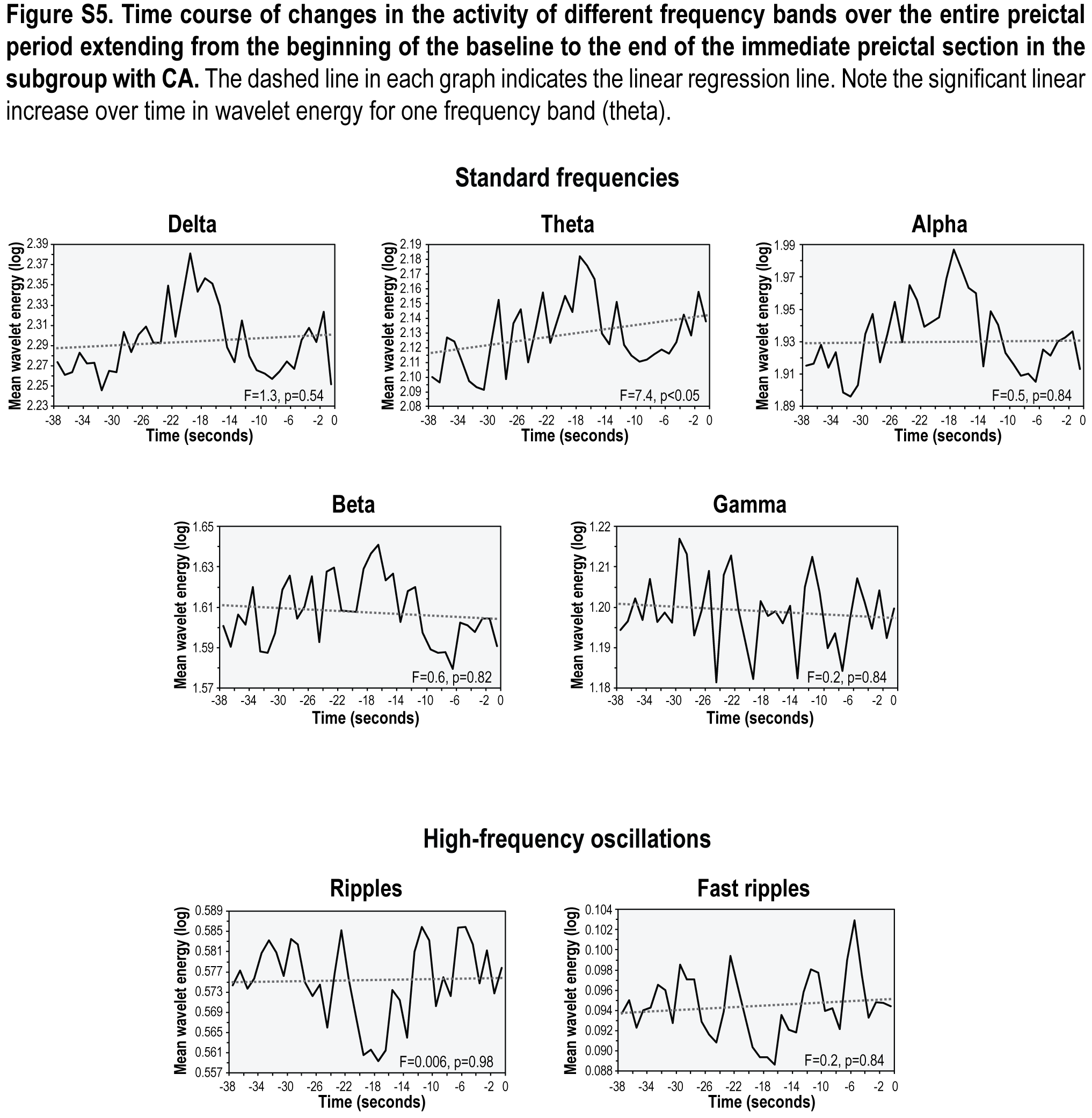

Supplement: Figure S5 — Time course of changes in the activity of different frequency bands over the entire preictal period extending from the beginning of the baseline to the end of the immediate preictal section in the subgroup with CA. The dashed line in each graph indicates the linear regression line. Note the significant linear increase over time in wavelet energy for one frequency band (theta). (TIF) [file pone.0080972.s005.tif]

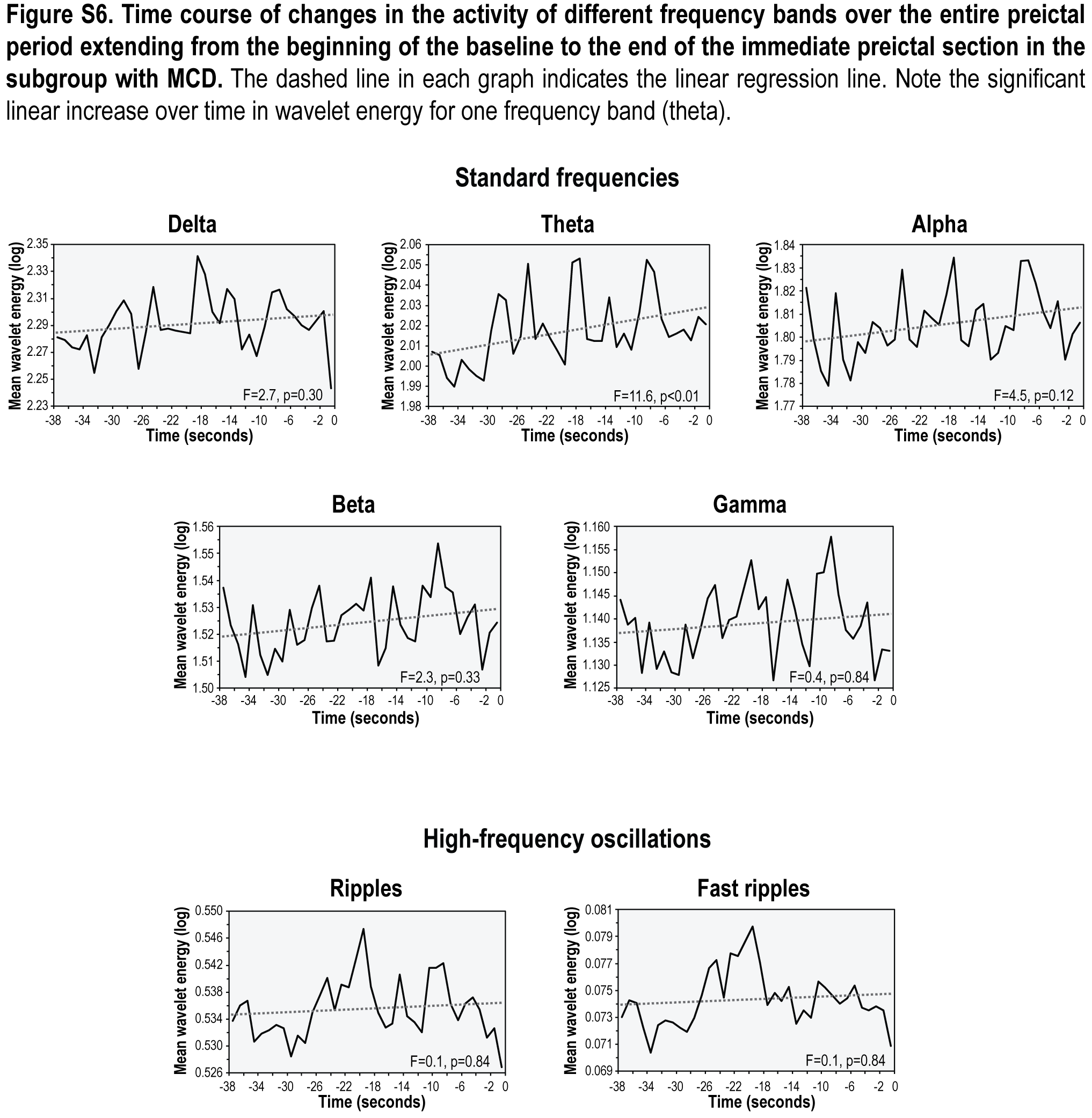

Supplement: Figure S6 — Time course of changes in the activity of different frequency bands over the entire preictal period extending from the beginning of the baseline to the end of the immediate preictal section in the subgroup with MCD. The dashed line in each graph indicates the linear regression line. Note the significant linear increase over time in wavelet energy for one frequency band (theta). (TIF) [file pone.0080972.s006.tif]
