# Supplementary material for: Widespread EEG Changes Precede Focal Seizures
Source: PLoS One. 2013 Nov 19;8(11):e80972. doi: 10.1371/journal.pone.0080972 (PMC3834227; doi:10.1371/journal.pone.0080972)
Supplement: Table S2 — Preictal changes in the activity of different frequency bands in each of the seizures included in the study (n = 70). Seizures have been stratified according to the type of significant preictal changes in frequency band activity: increases only (36 seizures), both increases and decreases (12 seizures), decreases only (10 seizures), and no significant change (12 seizures). (DOC) [file pone.0080972.s008.doc]

|  |  |  | **Percent change in frequency band activity from baseline to the immediate preictal section** | | | | | | |
| --- | --- | --- | --- | --- | --- | --- | --- | --- | --- |
| **Type of significant preictal changes in frequency band activity** | **Pt ID** | **Sz ID** | **Delta** | **Theta** | **Alpha** | **Beta** | **Gamma** | **Ripples** | **Fast ripples** |
| Increases only | 2 | 2 | 635.6%*** | 351.1%*** | 407.1%*** | 55.9%*** | 9.5% | 111.9% | 100.2% |
|  | 4 | 4 | 8.6% | 5.2% | -8.7% | -2.5% | 6.4%* | 14.0% | -17.2% |
|  |  | 5 | 243.8%*** | 396.6%*** | 186.4%*** | 84.4%*** | -0.2% | 6.4% | 3.5% |
|  | 5 | 6 | 35.9% | 73.3%** | 82.1%* | 45.4% | 42.2% | -19.2% | -53.4% |
|  | 6 | 7 | 51.5%*** | 48.9%*** | 77.1%*** | 32.7%** | 12.9%** | -32.9% | -7.3% |
|  | 8 | 11 | 23.8%* | 23.6%** | 10.4% | 26.2%** | 15.3%* | 62.0% | 243.8%* |
|  |  | 12 | 35.0%*** | 19.1%** | 35.6%*** | 51.2%*** | 32.1%*** | 16.5% | -36.4% |
|  | 10 | 14 | 67.8%** | 29.9%** | 30.5%** | 6.7% | 0.4% | 99.3% | -1.1% |
|  | 11 | 15 | 29.7%** | 18.7% | 1.2% | -10.5% | -3.5% | -5.6% | -53.5% |
|  | 12 | 17 | 50.1%*** | 35.7%** | 18.7% | 5.6% | 13.4%* | 97.3% | 2.5% |
|  | 13 | 18 | 13.8% | 19.1%** | 9.4% | 5.6% | 8.0%* | 8.6% | -29.0% |
|  |  | 20 | 21.1% | 22.9%*** | 22.2%* | 22.2%** | 3.2% | -37.9% | 10.1% |
|  |  | 21 | 270.5%*** | 201.7%*** | 68.2%* | 195.5%*** | 179.4%*** | 363.4%** | 43.5% |
|  | 14 | 22 | -1.3% | 33.9%* | 22.2% | 43.2%** | 3.1% | 13.4% | 5.4% |
|  | 15 | 26 | 41.6%*** | 29.2%*** | 24.5%*** | 20.4%*** | 9.9%*** | 58.8% | -21.4% |
|  | 16 | 27 | 35.5%** | 33.5%** | 2.7% | 0.9% | -0.7% | -29.9% | 15.8% |
|  | 22 | 36 | 27.8%** | 24.7%** | 49.5%*** | 50.4%*** | 45.3%*** | 248.3%** | 58.0% |
|  | 25 | 41 | 51.5%*** | 70.0%*** | 69.0%** | 116.4%*** | 149.2%** | 235.0%** | -10.6% |
|  | 26 | 42 | 39.4%** | 54.5%*** | 12.7% | -3.2% | 12.3%** | 101.9% | 256.4% |
|  | 28 | 45 | 261.4%*** | 202.3%*** | 70.9%*** | 57.9%*** | 29.7%* | 556.2%** | -30.9% |
|  |  | 46 | 31.4%* | 44.1%** | 5.2% | 14.9% | -10.0% | 488.9%** | -100.0% |
|  | 29 | 48 | 39.3% | 32.5%* | 0.03% | 6.8% | -12.2% | -4.0% | 83.3% |
|  |  | 49 | 97.0%*** | 184.3%*** | 115.8%*** | 42.5%*** | 7.3% | 260.6% | 58.9% |
|  |  | 50 | 34.8%* | 51.6%* | 24.8% | 40.1% | 108.8%** | 376.0%* | 249.5% |
|  | 31 | 53 | 14.4%* | 9.4% | -8.0% | 10.3% | -1.5% | -4.1% | -32.1% |
|  | 32 | 54 | 41.7%** | 48.9%** | -21.7% | 63.9%*** | 52.2%*** | 250.1%* | -4.2% |
|  |  | 55 | -6.7% | 10.0% | 4.0% | -4.2% | 16.2%** | 0.9% | 90.9% |
|  |  | 57 | 48.6%*** | 71.6%*** | 147.4%*** | 27.1%*** | -1.0% | 89.0% | 25.3% |
|  | 33 | 58 | 75.0%*** | 176.9%*** | 142.8%*** | 44.6%*** | 16.6%** | -1.1% | -69.3% |
|  | 35 | 61 | 4.6% | 54.9%*** | 59.5%*** | 14.0%* | -4.1% | 21.2% | -63.2% |
|  |  | 62 | 1.7% | 21.1% | 35.1% | 63.9%** | 113.6%*** | 730.9%*** | 692.0%** |
|  | 36 | 63 | 40.5%*** | 5.7% | 46.1%*** | 13.9%** | 5.6% | 250.6%** | 85.6% |
|  | 37 | 64 | 18.2%* | 11.5% | 19.2% | 33.3%*** | 27.8%*** | 72.1% | 10.8% |
|  | 38 | 66 | 9.1% | 14.8%* | 21.1%*** | -4.4% | -2.1% | -40.2% | n/c |
|  |  | 67 | 80.5%*** | 164.8%*** | 61.1%*** | -23.3%** | -22.2%** | -6.7% | 83.7% |
|  | 39 | 68 | 8.1% | 10.1% | 18.4% | 30.5%** | 18.8%* | 179.3%* | 179.5% |
| Both increases and decreases | 1 | 1 | 180.3%*** | 55.7%*** | 19.3%* | -15.8%* | -9.8%* | -9.4% | 113.3% |
|  | 3 | 3 | -18.5% | -34.9%** | 4.6% | 15.5% | 32.3%** | 70.4% | -32.1% |
|  | 7 | 10 | 77.3%*** | 40.7%** | 22.4%* | 7.2% | -13.1%** | 56.6% | 45.0% |
|  | 9 | 13 | 18.6%* | 7.5% | 1.4% | -10.2% | -10.6%* | 17.0% | -18.7% |
|  | 11 | 16 | 111.2%*** | 31.8%** | -29.4%** | -34.2%*** | -21.9%** | 36.2% | -17.6% |
|  | 17 | 30 | 124.7%*** | 100.9%*** | 58.0%*** | 15.9%* | -19.3%*** | 63.0% | -63.1% |
|  | 23 | 37 | 22.8%* | 15.5% | 42.1%** | 24.7%*** | 0.4% | -58.5%* | -100.0% |
|  | 26 | 43 | -1.9% | -24.7%** | -13.4% | -0.2% | 5.7%* | 17.8% | n/c |
|  | 29 | 47 | -24.5%** | -16.7%* | -15.8% | 12.7%* | 13.3%* | -13.1% | 86.8% |
|  | 30 | 51 | -21.3%** | 20.4%** | 8.8% | -1.7% | 3.3% | -28.6% | -57.6% |
|  | 34 | 59 | -21.9%*** | -6.5%* | -1.5% | 15.9%*** | 8.1%* | -65.3%* | -73.5% |
|  | 39 | 69 | 131.1%*** | 21.9% | -11.3% | -26.4%** | 9.2%* | 23.4% | 67.8% |
| Decreases only | 13 | 19 | -33.3%*** | -24.6%*** | -23.2%*** | -41.5%*** | -41.8%*** | -6.9% | -8.6% |
|  | 17 | 28 | -10.0% | -3.8% | -23.2%** | -10.2% | 0.03% | -1.7% | -14.8% |
|  | 18 | 31 | -36.5%*** | -13.3% | -30.4%** | 9.1% | -11.9% | -28.2% | -39.7% |
|  | 20 | 33 | 14.9% | -9.8% | -31.6%** | -3.0% | 16.1% | 85.2% | n/c |
|  | 21 | 34 | -37.6%*** | -13.9% | -3.1% | 1.2% | 6.0% | -18.8% | 98.2% |
|  |  | 35 | -16.5%* | -22.3%*** | -48.9%*** | -15.0%** | 5.0% | 62.7% | 46.3% |
|  | 24 | 39 | -18.6%* | -25.0%*** | -26.1%*** | -27.0%*** | -12.6%*** | -34.5% | -42.8% |
|  | 25 | 40 | -60.5%*** | -48.0%** | -18.6%* | -26.9%* | -8.0% | -38.1% | -55.3% |
|  | 32 | 56 | 1.5% | -4.2% | -42.7%*** | -17.7%** | 3.7% | 155.8% | n/c |
|  | 35 | 60 | -45.1%*** | -57.9%*** | -21.1%* | -30.2%*** | -2.7% | 245.5% | 234.8% |
| No significant changes | 6 | 8 | 13.5% | -14.2% | -24.1% | -0.5% | 8.3% | 69.1% | 57.1% |
|  |  | 9 | 6.7% | -7.6% | -11.4% | -8.0% | -0.7% | 122.5% | -8.4% |
|  | 14 | 23 | 23.8% | 14.8% | 15.0% | 3.3% | 10.6% | -34.9% | -3.0% |
|  | 15 | 24 | 9.2% | -2.9% | -7.7% | -3.1% | 6.8% | 36.1% | -56.6% |
|  |  | 25 | -14.7% | -13.5% | -15.8% | 10.3% | 0.2% | -3.1% | 18.1% |
|  | 17 | 29 | 4.0% | -13.6% | -17.7% | -6.0% | 0.1% | 61.7% | -37.3% |
|  | 19 | 32 | 3.7% | 13.4% | 5.4% | 4.4% | -0.2% | -19.1% | 3.4% |
|  | 23 | 38 | 5.2% | 0.4% | -9.8% | -3.9% | 0.7% | -16.2% | -60.3% |
|  | 27 | 44 | 18.0% | -3.5% | 3.9% | 1.2% | 1.4% | -0.6% | -31.8% |
|  | 30 | 52 | 6.0% | -13.6% | -20.6% | -28.6% | 7.4% | 61.3% | 128.5% |
|  | 37 | 65 | 0.6% | -0.4% | 2.9% | 10.0% | -1.3% | 6.0% | -17.5% |
|  | 40 | 70 | -10.8% | -13.0% | -11.3% | -5.3% | -6.8% | 118.7% | n/c |

***p<0.001; **p<0.01; *p≤0.05

Pt ID=patient identification number; Sz ID=seizure identification number; n/c=not computable due to lack of fast ripples in baseline
