# Supplementary material for: Widespread EEG Changes Precede Focal Seizures
Source: PLoS One. 2013 Nov 19;8(11):e80972. doi: 10.1371/journal.pone.0080972 (PMC3834227; doi:10.1371/journal.pone.0080972)
Supplement: Table S3 — Comparison of the activity of different frequency bands between the baseline section and the immediate preictal section in the three patient subgroups, shown for the entire set of contacts and for four separate contact subsets (lesional/SOZ, non-lesional/SOZ, lesional/non-SOZ, non-lesional/non-SOZ). For standard frequency bands, values in cells are mean spectral power with 95% confidence interval (95% CI). For HFOs, values in cells are mean percentage of time occupied by HFOs in each section (95% CI). (DOC) [file pone.0080972.s009.doc]

|  |  | **Frequency band activity in the two sections stratified by the underlying pathology** | | | | | |
| --- | --- | --- | --- | --- | --- | --- | --- |
|  |  | **Mesial temporal atrophy/sclerosis** | | **Local/regional cortical atrophy** | | **Malformations of cortical development** | |
| **Set of contacts** | **Frequency band** | ***Baseline*** | ***Immediate preictal*** | ***Baseline*** | ***Immediate preictal*** | ***Baseline*** | ***Immediate preictal*** |
| All contacts^a^ | Delta | 186.2 (165.2-209.8) | 234.1 (206.3-265.7)*** | 209.5 (180.2-243.4) | 243.9 (210.0-283.2)*** | 195.6 (174.5-219.2) | 238.4 (212.8-267.1)*** |
|  | Theta | 91.6 (81.6-102.9) | 115.3 (102.4-129.9)*** | 131.7 (114.2-151.8) | 144.4 (125.3-166.4)** | 65.9 (59.4-73.1) | 76.9 (69.0-85.7)*** |
|  | Alpha | 51.2 (45.5-57.7) | 65.2 (57.8-73.6)*** | 74.9 (64.8-86.5) | 75.9 (65.9-87.3) | 39.1 (35.0-43.7) | 41.9 (37.4-47.0)*** |
|  | Beta | 35.4 (31.3-39.9) | 40.7 (36.0-46.1)*** | 57.8 (49.6-67.3) | 55.3 (47.8-63.9)* | 38.6 (34.4-43.4) | 41.5 (36.9-46.6)*** |
|  | Gamma | 6.5 (5.8-7.3) | 7.2 (6.4-8.2)*** | 9.9 (8.6-11.5) | 10.3 (8.9-11.9)** | 7.3 (6.5-8.1) | 7.5 (6.7-8.3)** |
|  | Ripples | 0.025 (0.019-0.032) | 0.043 (0.034-0.055)*** | 0.029 (0.021-0.039) | 0.037 (0.028-0.050)* | 0.027 (0.022-0.033) | 0.033 (0.026-0.040)* |
|  | Fast ripples | 0.008 (0.006-0.010) | 0.011 (0.008-0.014)** | 0.007 (0.005-0.010) | 0.007 (0.005-0.010) | 0.006 (0.004-0.007) | 0.006 (0.005-0.008) |
| Lesional/SOZ contacts^b^ | Delta | 463.4 (301.7-711.8) | 579.1 (371.7-902.3)** | 694.8 (352.6-1369.2) | 765.1 (347.6-1684.0) | 521.4 (383.5-708.8) | 627.4 (460.1-855.4)* |
|  | Theta | 170.9 (110.0-265.6) | 237.7 (153.3-368.7)*** | 274.1 (138.2-543.6) | 266.1 (137.7-514.3) | 137.3 (104.4-180.7) | 173.5 (130.5-230.7)*** |
|  | Alpha | 87.3 (54.1-140.9) | 119.4 (76.8-185.6)*** | 103.4 (53.9-198.4) | 86.4 (48.4-154.2) | 71.3 (54.2-93.8) | 84.1 (64.3-110.0)** |
|  | Beta | 75.4 (47.9-118.7) | 100.0 (63.1-158.7)** | 60.9 (34.1-108.8) | 54.6 (33.2-89.8) | 51.2 (37.9-69.2) | 60.1 (44.7-81.0)*** |
|  | Gamma | 17.4 (11.2-27.0) | 20.6 (13.3-31.9)* | 12.2 (6.9-21.7) | 12.2 (7.1-21.0) | 11.2 (8.5-14.7) | 13.0 (9.8-17.3)** |
|  | Ripples | 0.316 (0.139-0.703) | 0.664 (0.337-1.301)** | 0.117 (0.035-0.353) | 0.165 (0.049-0.508) | 0.066 (0.038-0.111) | 0.092 (0.053-0.156) |
|  | Fast ripples | 0.112 (0.048-0.247) | 0.289 (0.137-0.599)** | 0.015 (0.001-0.045) | 0.009 (0.0001-0.027) | 0.019 (0.010-0.030) | 0.020 (0.012-0.032) |
| Non-lesional/SOZ contacts^c^ | Delta | 311.8 (200.0-485.9) | 398.7 (247.5-642.3)** | 677.9 (278.1-1652.0) | 781.2 (343.5-1776.5) | 584.1 (356.4-957.3) | 628.3 (410.2-962.4) |
|  | Theta | 130.2 (85.8-197.7) | 170.1 (109.4-264.4)** | 315.4 (158.6-627.3) | 341.8 (169.9-687.6) | 174.4 (116.3-261.6) | 206.5 (138.2-308.4)* |
|  | Alpha | 49.5 (32.5-75.3) | 68.7 (42.9-110.0)*** | 127.1 (65.8-245.3) | 144.3 (72.0-289.0) | 98.2 (65.0-148.3) | 102.0 (67.9-153.2) |
|  | Beta | 42.1 (27.9-63.5) | 55.6 (34.6-89.3)** | 118.4 (62.5-224.2) | 150.6 (75.6-300.0) | 79.3 (52.9-118.9) | 89.6 (59.9-134.1) |
|  | Gamma | 10.9 (7.2-16.4) | 14.5 (9.5-22.1)** | 35.3 (17.1-72.8) | 43.6 (20.6-92.5)* | 21.5 (14.0-33.0) | 22.4 (14.6-34.4) |
|  | Ripples | 0.078 (0.030-0.181) | 0.124 (0.051-0.285) | 0.235 (0.058-0.864) | 0.257 (0.068-0.902) | 0.090 (0.033-0.0220) | 0.133 (0.053-0.314) |
|  | Fast ripples | 0.041 (0.017-0.084) | 0.039 (0.017-0.080) | 0.057 (0.016-0.163) | 0.050 (0.013-0.145) | 0.012 (0.004-0.026) | 0.015 (0.005-0.031) |
| Lesional/non-SOZ contacts^d^ | Delta | 226.1 (133.9-381.8) | 345.5 (204.9-582.4)*** | 190.4 (146.4-247.6) | 220.6 (168.5-288.7)** | 140.6 (107.4-184.1) | 170.9 (130.8-223.4)** |
|  | Theta | 87.8 (53.5-144.1) | 139.7 (87.8-222.4)** | 122.8 (94.5-159.5) | 137.7 (104.6-181.3) | 49.8 (38.1-65.0) | 57.0 (43.7-74.2)* |
|  | Alpha | 50.2 (30.1-83.8) | 69.3 (41.3-116.2)** | 66.3 (48.9-90.0) | 61.9 (45.9-83.4) | 33.6 (25.1-45.1) | 34.4 (25.4-46.4) |
|  | Beta | 38.2 (23.5-62.2) | 47.9 (29.6-77.7)** | 54.1 (38.8-75.5) | 47.0 (34.5-64.1)** | 24.6 (18.4-32.8) | 25.8 (19.2-34.6) |
|  | Gamma | 8.5 (5.4-13.3) | 10.0 (6.5-15.3) | 8.4 (6.2-11.4) | 8.8 (6.5-11.8) | 4.7 (3.6-6.2) | 4.8 (3.7-6.4) |
|  | Ripples | 0.053 (0.018-0.135) | 0.139 (0.055-0.332) | 0.021 (0.011-0.036) | 0.025 (0.014-0.043) | 0.027 (0.015-0.045) | 0.036 (0.021-0.058) |
|  | Fast ripples | 0.022 (0.006-0.051) | 0.031 (0.009-0.075) | 0.007 (0.003-0.012) | 0.008 (0.004-0.013) | 0.006 (0.003-0.011) | 0.007 (0.003-0.012) |
| Non-lesional/non-SOZ contacts^e^ | Delta | 153.9 (135.3-175.0) | 190.3 (165.9-218.3)*** | 172.7 (144.3-206.6) | 202.9 (170.4-241.6)*** | 148.8 (130.5-169.7) | 184.4 (161.4-210.7)*** |
|  | Theta | 81.6 (71.8-92.6) | 99.1 (87.0-112.9)*** | 115.9 (97.2-138.2) | 127.4 (107.3-151.4)** | 53.3 (47.2-60.3) | 61.2 (53.8-69.7)*** |
|  | Alpha | 48.2 (42.2-55.0) | 59.8 (52.3-68.4)*** | 72.8 (61.3-86.6) | 77.4 (65.6-91.5) | 31.9 (27.8-36.5) | 33.9 (29.4-38.9)* |
|  | Beta | 31.3 (27.4-35.9) | 34.7 (30.3-39.6)*** | 55.3 (46.0-66.3) | 54.2 (45.6-64.4) | 37.6 (32.5-43.4) | 39.5 (34.1-45.6)** |
|  | Gamma | 5.3 (4.7-6.1) | 5.7 (5.0-6.5)*** | 9.3 (7.8-11.0) | 9.5 (8.0-11.3) | 6.5 (5.7-7.5) | 6.5 (5.7-7.4) |
|  | Ripples | 0.013 (0.009-0.017) | 0.022 (0.016-0.029)*** | 0.023 (0.015-0.033) | 0.030 (0.020-0.043) | 0.018 (0.014-0.023) | 0.020 (0.015-0.025) |
|  | Fast ripples | 0.002 (0.001-0.003) | 0.003 (0.002-0.004) | 0.005 (0.002-0.007) | 0.004 (0.002-0.007) | 0.003 (0.002-0.004) | 0.003 (0.002-0.004) |

***p<0.001; **p<0.01; *p≤0.05

^a^ Mesial temporal atrophy/sclerosis (n=562), local/regional cortical atrophy (n=392), malformations of cortical development (n=793); ^b^ Mesial temporal atrophy/sclerosis (n=53), local/regional cortical atrophy (n=24), malformations of cortical development (n=126); ^c^ Mesial temporal atrophy/sclerosis (n=51), local/regional cortical atrophy (n=23), malformations of cortical development (n=48); ^d^ Mesial temporal atrophy/sclerosis (n=33), local/regional cortical atrophy (n=111), malformations of cortical development (n=125); ^e^ Mesial temporal atrophy/sclerosis (n=425), local/regional cortical atrophy (n=234), malformations of cortical development (n=494)
